# *In vivo* online magnetic resonance quantification of absolute metabolite concentrations in microdialysate

**DOI:** 10.1038/srep36080

**Published:** 2016-11-04

**Authors:** Stefan Glöggler, Silvia Rizzitelli, Noël Pinaud, Gérard Raffard, Vanessa Zhendre, Véronique Bouchaud, Stéphane Sanchez, Guillaume Radecki, Luisa Ciobanu, Alan Wong, Yannick Crémillieux

**Affiliations:** 1 ISM, UMR 5255, Université Bordeaux, 33076, Bordeaux, France; 2 RMSB, UMR 5536, Université Bordeaux, 33076, Bordeaux, France; 3 CEA I2BM NeuroSpin, 91191, Gif-sur-Yvette, France; 4 NIMBE, CEA, CNRS, Université Paris-Saclay, CEA Saclay, 91191, Gif-sur-Yvette, France

## Abstract

In order to study metabolic processes in animal models of diseases and in patients, microdialysis probes have evolved as powerful tools that are minimally invasive. However, analyses of microdialysate, performed remotely, do not provide real-time monitoring of microdialysate composition. Microdialysate solutions can theoretically be analyzed online inside a preclicinal or clinical MRI scanner using MRS techniques. Due to low NMR sensitivity, acquisitions of real-time NMR spectra on very small solution volumes (μL) with low metabolite concentrations (mM range) represent a major issue. To address this challenge we introduce the approach of combining a microdialysis probe with a custom-built magnetic resonance microprobe that allows for online metabolic analysis (^1^H and ^13^C) with high sensitivity under continuous flow conditions. This system is mounted inside an MRI scanner and allows performing simultaneously MRI experiments and rapid MRS metabolic analysis of the microdialysate. The feasibility of this approach is demonstrated by analyzing extracellular brain cancer cells (glioma) *in vitro* and brain metabolites in an animal model *in vivo*. We expect that our approach is readily translatable into clinical settings and can be used for a better and precise understanding of diseases linked to metabolic dysfunction.

Microdialysis is a valuable tool for *in vivo* investigations and can be placed in many tissues and organs including brain for neurological investigations of Alzheimer^1^,^2^, brain tumors^3^, traumatic brain injuries^4^, neuronal functionality^5^ and pharmacokinetics of drugs^6^. In combination with magnetic resonance imaging (MRI), dialysate composition can be directly correlated with anatomical structures or pathological changes seen on MRI^7^,^8^. However, metabolic investigations of the dialysate are currently performed remotely and off-line from the MRI scanner^9^,^10^,^11^,^12^ due to the limited online sampling possibilities^13^,^14^. Integrating analytical devices within MRI scanners poses challenges (related to limited space and to magnetic interference), limiting the applications of correlation experiments. Here, we introduce a novel approach of online sampling of tissue dialysate under continuous flow utilizing a high resolution nuclear magnetic resonance (NMR) microprobe, closely coupled to an *in vivo* microdialysis inside an MRI scanner. We present two microprobes that allow for the online metabolic investigation at physiological concentrations (^1^H: 1.8 mM lactate in 60 s; ^13^C: 10.0 mM 3-^13^C lactate in 100 s). The applicability of the approach is demonstrated by investigating extracellular metabolites, from *in vitro* brain cancer cells and from *in vivo* microdialysate of rat brain.

## Results

Micro-structured NMR/MRS probes have been fabricated in a variety of different geometries^15^,^16^,^17^,^18^,^19^,^20^,^21^,^22^,^23^,^24^,^25^,^26^,^27^,^28^ and have partly been applied to continuous flow investigations^29^,^30^ with solenoidal coils being the most sensitive to date^15^,^16^. Utilizing this fact with the goal of online detection of metabolites at physiological concentrations for *in vivo* studies, we have constructed two different microprobes with solenoidal geometry: a ^1^H-optimized probe tuned at 300 MHz and a ^13^C-optimized probe with unprecedented sensitivity tuned at 50 MHz.

**Figure 1:**
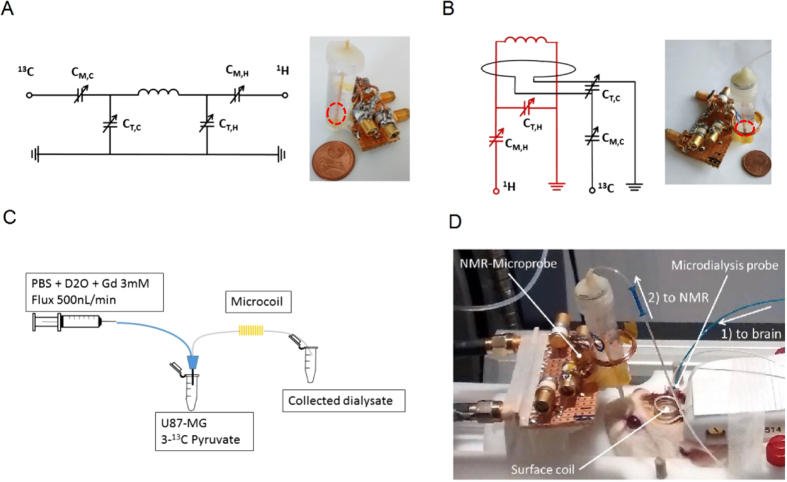
Experimental setups. (**A**) Left: Circuit of the ^1^H/^13^C double resonant ^13^C-optimized probe for carbon detection at 50 MHz, right: photo illustration of the microprobe (red circles indicate the position of the microcoil). (**B**) Left: Circuit of the ^1^H- optimized probe for proton detection at 300 MHz with a separate ^13^C decoupling channel (75 MHz), right: photo illustration of the microprobe. (**C**) *In vitro* experiment utilizing a microprobe/microdialysis setup to collect and analyze extracellular glioma metabolites under a continuous flow. (**D**) *In vivo* setup to collect brain metabolites from a rat including the possibility of acquiring images of the brain with a surface proton coil (arrows indicate the flow of the buffer solution/dialysate).

The proton and carbon-13 microcoils are respectively interfaced and positioned inside MRI scanners operating at B_0_ = 7.0 T and B_0_ = 4.7 T (Fig. 1A,B), respectively. For both probes, the detected sample volume is 1 μL (2.4 mm in length and 750 μm in diameter) with an optimal filling factor of 92.5% (the ratio of the sample volume to the coil detection volume). The limits of detection (LOD) for the probes, corresponding to a signal-to-noise ratio (SNR) of 3, are 1.8 nmol in 60 s for the methyl protons (which corresponds to 5.4 nmol for one proton) of lactate and 10 nmol in 100 s for the carbon atoms in an enriched 3-^13^C lactate sample. Characteristics of the coils are summarized in Table 1 whereby the detection sensitivity has been normalized according to an established procedure (see ref. [25], equation 5 and Table 1) to a field strength of B_0_ = 14.1 T. The sensitivities amount to 12.7 nmol·s^½^ for the proton-optimized probe and 14.6 nmol·s^½^ for the carbon-optimized coil. The spectral resolution is optimized by placing the microcoil into a container filled with fluorinated solvent for increasing the magnetic field homogeneity at the site of the sample. The linewidths for the ^1^H- (B_0_ = 7 T) and ^13^C-optimized (B_0_ = 4.7 T) probes were determined using NMR lines from natural abundance and ^13^C-enriched lactate, respectively leading to proton linewidth of 15.0 Hz (0.05 ppm) and carbon linewidth of 3.5 Hz (0.07 ppm) inside the MRI scanners. The broadened line width can be explained by the increased susceptibility mismatch due to the proximity of the sample to the windings of the copper coil as has been previously investigated by Webb and coworkers^31^. Since we optimized the filling factor to over 90% we achieve a near optimal SNR but compromise the line-resolution. For ^13^C NMR experiments, a double resonant circuit, based on a single ^13^C-^1^H micro-size solenoid is used in transmit-receive mode (Fig. 1A). The circuit is optimized for ^13^C detection while the ^1^H channel can be used for the shimming procedure (homogenizing the magnetic field in the sample region). In addition, the ^1^H channel can also be utilized for proton decoupling in ^13^C experiments to further enhance the sensitivity, this being particularly useful for a ^13^C-enriched dialysate solution. Unlike the ^13^C microprobe, the ^1^H probe consists of two independent resonance circuits (Fig. 1B): a micro-solenoid is used for ^1^H excitation, detection and B_0_ shimming; the second resonant circuit, composed of a 1.5-cm diameter loop, is used as a ^13^C decoupling channel.

The experimental setup for online *in vitro* and *in vivo* MR analysis of microdialysate is depicted in Fig. 1C,D. Inside the MRI scanner, a microdialysis probe is inserted in an Eppendorf tube containing cells or positioned inside the target’s brain region. The outlet-tube from the microdialysis is immediately passed through the microprobe for a real-time online metabolic profiling of the dialysate. Outside the scanner, the inlet-tube is connected to a syringe pump that continuously flows a buffer solution through the sample i.e., cells or brain.

**Figure 2:**
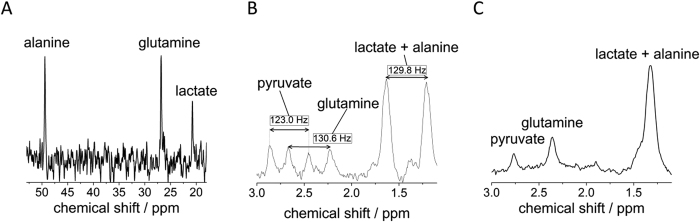
*In vitro* experiments on glioma cells. (**A**) ^13^C spectrum of extracellular glioma metabolites in the dialysate after the addition of enriched 3-^13^C-pyruvate; acquired under continuous flow in 1000 s at B_0_ = 4.7 T (50 MHz). (**B**) ^1^H spectrum of extracellular glioma metabolites acquired after the addition of 3-^13^C-pyruvate at B_0_ = 7 T (300 MHz). The ^1^H-^13^C J-splitting shown in the spectrum indicates that the MRS-detected metabolites are originated from the supplied 3-^13^C-labeled pyruvate. (**C**) ^13^C decoupled spectrum shows the isotropic chemical shift signals of the metabolites from the microdialysate (differences in chemical shift compared to (**B**) are due to the Bloch-Siegert shift upon a strong decoupling field).

In the *in vitro* study, we attempt to monitor the metabolism activities of cancer cells (glioma) supplied with 3-^13^C pyruvate. As shown in Fig. 1C, the microdialysis is placed inside a buffer solution containing U87-MG glioma cells. The MRS (magnetic resonance spectroscopy) acquisition begins as the 3-^13^C pyruvate is added to the suspension of cells. The MRS results from the subsequent sampling with the ^13^C and ^1^H microprobes are depicted in Fig. 2. With the ^13^C-optimized microprobe (Fig. 2A), signals for ^13^C labeled alanine, glutamine and lactate are detected in less than 17 minutes (B_0_ = 4.7 T) whereby most of the pyruvate has already been metabolized as confirmed by high-field spectroscopy experiments (see Methods section). The LOD for a measurement of ^13^C-enriched metabolites on a 17-minute timescale is 3.2 mM (i.e. 3.2 nmol in 1 μL), with a measured linewidth of 5.5 Hz. The same experiment was performed at B_0_ = 7 T with the ^1^H probe (Fig. 2B) by averaging over less than 4 minutes, and resulted in a LOD of 0.9 mM (900 pmol in 1 μL) for three protons of the methyl group of the ^13^C-enriched metabolites lactate, alanine and pyruvate (linewidth of 20.0 Hz) and an LOD of 1.4 mM for the two glutamine protons. Based on these LODs, single proton species are expected to be detectable if the concentration exceeds 2.7 mM (2.7 nmol in 1 μL). We note that alanine and lactate signals could be better distinguishable from each other with an improved linewidth; the existence of both metabolites was however confirmed by high field NMR spectroscopy at B_0_ = 11.7 T (see Methods information).

Differentiation between extracellular metabolites originating from the ^13^C enriched pyruvate substrate and other endogenous substrates can be performed (1) by observing J-splitting in the presence of ^1^J_H,C_ coupling, and (2) by performing a subsequent ^13^C decoupling experiment after which only one singlet becomes visible for the ^13^C-enriched metabolites. For example, the spectrum from a ^1^H single-pulse experiment, Fig. 2B, displays the J-splitting for ^13^C-enriched pyruvate, glutamine, lactate and alanine. For residual pyruvate a J-splitting of ^1^J_H,C_ ≈ 123 Hz was observed and for glutamine ^1^J_H,C_ ≈ 130.6 Hz. The splitting of the “combined” lactate and alanine species corresponds to 129.8 Hz. Conversely, only isotropic chemical shift signals are observed in the ^13^C-decoupled experiment in Fig. 2C. Moreover, the direct ^13^C detection from ^13^C experiment also exhibits three distinct metabolite signals. These ^1^H and ^13^C spectroscopic results clearly confirm the presence the ^13^C-enriched metabolites in the dialysate; and thus, they indicate that the infused ^13^C-pyruvate has been metabolized by the cells.

**Figure 3:**
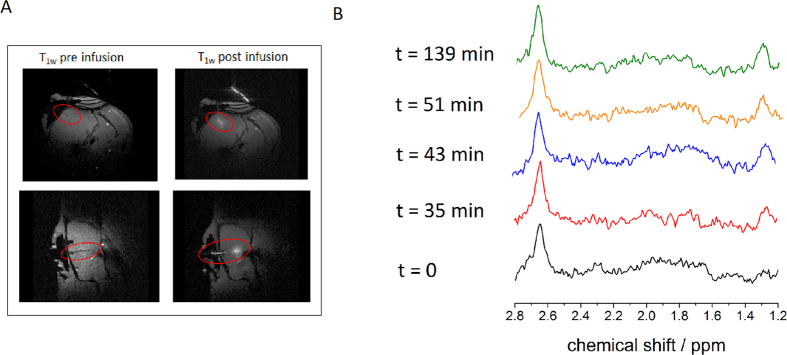
*In vivo* experiments. (**A**) *T*_1_-weighted MR images in axial (top) and sagittal (bottom) planes of microdialysis probe positioning before (left images) and after (right images) flowing the perfusate. The bright area demonstrates the diffusion of Gd-DOTA through the microdialysis membrane into the rat brain. (**B**) Time course of the spectroscopy experiments with the ^1^H-optimized microprobe at B_0_ = 7 T. The bottom line is the starting spectrum at t = 0 in which only the meglumine standard (left peak at 2.7 ppm) is visible. After t = 35 min lactate becomes observable (right peak at 1.3 ppm), the equilibrium is reached after 43 minutes (i.e. no increase in lactate concentration is visible over the course of a 139-minute experiment) and 1.1 mM lactate is detected in the dialysate under continuous flow and 3 minutes and 50 seconds of NMR acquisition.

In the *in vivo* study on a rat model, the microdialysis was inserted into the cortex of the rats’ brain via a guide cannula and connected to the inlet of the ^1^H probe. Under continuous flow with buffer solution that included 3 mM of DOTAREM^®^ (Gd-DOTA and meglumine), the dialysate brain metabolites were analyzed. The gadolinium contrast agent serves three purposes: (1) monitoring the activity of the microdialysis inside the rat brain using MRI (Fig. 3A); (2) shortening the ^13^C longitudinal relaxation time for faster NMR acquisition; and (3) serving as an internal reference (the line of the meglumine methyl ^1^H appears at 2.8 ppm) for the NMR chemical shift as well as for the concentration. Fig. 3B depicts the time course for the *in vivo* experiments. In the beginning, when the perfusate flow is started, only protons of water and protons from meglumine are detected by the microcoil. The dialysate reaches the microcoil after 35 minutes and the extracellular brain metabolites become detectable at 1.2 ppm in the ^1^H NMR spectrum (0.7 mM concentration). The 35 minutes delay is due to the approximately 18 μL volume of tubing placed between the rat and the RF coil. This delay can be significantly reduced using shorter tubing with a smaller inner diameter. We expect that with an optimized tubing the volume can be reduced down to 1 μL which will allow for acquiring the first spectrum within a few minutes, making it more practical for *in vivo* use with patients. After 43 minutes the equilibrium concentration of lactate from the rat brain can be detected, which stays constant over the course of the experiment (139 minutes). The comparison with the meglumine standard reveals that a concentration of 1.1 mM extracellular lactate from the brain is detected at equilibrium. The existence of the lactate metabolite was further proven by high-field NMR spectroscopy (see Methods section).

With respect to translatability to clinical patients, we see great potential in combination with ultrahighfield MRI systems for which wholebody scanners up to 7T exist^32^. Although we present in our article the study of the brain in animal models, we would like to note that the use of microdialysis in connection with clinical patients is possible in many different organs. For examples, microdialysis probes have been applied to breast cancer research, and to liver, adipose tissue or skeletal muscle *in vivo* studies of patients^33^,^34^,^35^. Furthermore, clinical studies to probe pharmacokinetics and visualization with gadolinium contrast agents have been performed previously. However with the approach present here, it is now possible to carry out the measurement inside the MRI scanner without the necessity of performing the analysis at a remote place^36^. An advantage with clinical patients is that larger microdialysis membranes are used for sampling^37^. Thus, higher amounts of sample can be analyzed in slightly larger NMR microprobes which will further increase the SNR. Lastly, we expect that the microprobes are not only combinable with surface coil but could also be used together with volume coils. In this case, care has to be taken that the microprobe is still mounted inside a homogeneous region of the MRI system but far enough away from the imaging region to prevent artifacts.

We expect that the online MR metabolic analysis of microdialysate presented in this study opens new perspectives for metabolite profiling. The particular strength of the approach lies in the possibility to perform metabolic analysis in rapid succession of imaging experiments on a timescale of 3 to 20 minutes in order to investigate the metabolic evolution under chemiotherapeutic treatment or neural activity. As proof-of-principle *in vivo* experiments, we have successfully detected highly concentrated lactate (1.1 mM in the dialysate) with the microcoil. Lower concentrated metabolites are expected to become detectable with lower flow rates allowing for an improved exchange of metabolites into the membrane and by improving the microprobe. Further increases in resolution and sensitivity can be expected by utilizing higher order shims, by increasing the microcoil volume or by building microcoils for MRI scanners operating at higher magnetic fields.

The application of this combined microprobe and microdialysis systems is not only limited to the brain study, but it can also be implemented in other organs or living tissues. As microdialysis probes are being used *in vivo* in humans^4^ and microcoil probes are being utilized for human exploration^18^, we further expect that the MR approach presented here could be applied in clinical settings.

## Methods

### Microcoil design

The double-parallel microcoils (length = 2.4 mm) were handwound with 30 μm (^13^C probe, 80 windings) or 62.5 μm (^1^H probe, 38 windings) copper wire around a polyimide tubing (inner diameter = 750 μm and outer diameter = 780 μm) that was supported during the winding process by a glass capillary and was removed afterwards. The lower and upper end of the coil are supported by a larger polyimide tubing (inner diameter = 800 μm and outer diameter = 858 μm), eliminating the necessity of using glue, which may compromise the probe’s quality, for coil fixation. The coils were placed inside a 2 ml Eppendorf tube which was filled with FC-70 fluid.

### Chemicals

Sodium L-lactate-3-^13^C solution, Sodium pyruvate-3-^13^C, D_2_O and all the inorganic salts were purchased from Sigma-Aldrich (St Louis, MO, USA), and used without further purification. Dotarem^®^ was provided by Guerbet Group (Villepinte, France). The culture medium DMEM, the biological buffers, foetal calf serum (FCS), penicillin-streptomycin mixture, and trypsin were purchased from Gibco Corp - Thermo Fisher Scientific (Waltham, MA, USA).

### Cell culture

Human glioblastoma U87-MG cell line, purchased by ATCC (LGC standards, Middlesex, UK), were cultured as monolayer at 37 °C in a 5% CO_2_-containing humidified atmosphere. The culture medium was composed of Dulbecco’s modified eagle medium (DMEM low glucose content and contains L-glutamine, reference 31885) supplemented with 10% (v/v) heat-inactivated FCS, 100 IU/mL penicillin, and 100 IU/mL streptomycin.

### Experimental cell setup

Cells were separated from DMEM by centrifugation and washing with PBS buffer prior to the experiment. 7 × 10^6^ cells were suspended in 300 μL of PBS buffer within an Eppendorf tube. With the aim of keeping the cells in suspension, a plastic tubing connected to an infusion pump for air injection was inserted into the cells solution leading to continuous stirring. The cell suspension was heated to 37 °C in all experiments with external circulating warm water. The temperature was checked with a thermocouple. No temperature induced shift in the resonances was observed during the experiments. A microdialysis probe was placed into the cells suspension and infused with a buffer solution (PBS in D_2_O containing 3 mM Dotarem^®^) under a flux of 500 nL/min with the use of an infusion pump (Legato 110, Phymep, Paris, France). The outlet tube of the microdialysis probe was inserted into the microcoil tube to analyse the dialysate by MR spectroscopy. 3–13C sodium pyruvate solution was added to yield an overall concentration of 150 mM. The experiment was continuously performed for 2 hours.

**Figure 4:**
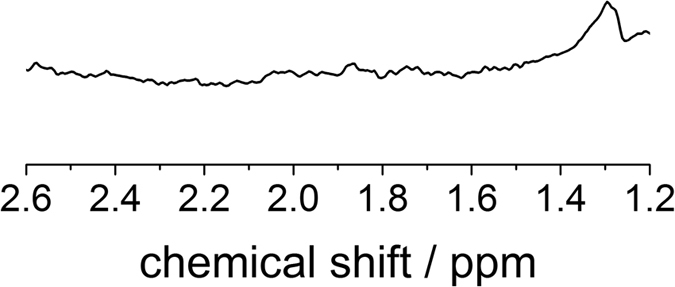
^1^H spectrum of an *in vitro* control experiment. Upon the absence of 3-^13^C pyruvate no ^13^C enriched source is detected. Lactate in natural abundance from an internal source is detected which is identified in the high field spectrum as well during the 13C experiments (see Fig. 5). However no other metabolites are identifiable in this control experiment.

Additionally, a control experiment without ^13^C-pyruvate was performed (Fig. 4).

### Animal model

Adult female Wistar rats of 250 g weights were purchased from Charles River Laboratories and kept in standard housing (12 h light-dark cycles) with a standard rodent chow and water available ad libitum. Before the beginning of the experiment, the animals were acclimatized in a temperature-controlled environment for one week. Experiments were performed according to the national regulations and were approved by the research ethics committee of the University of Bordeaux. The animals were anesthetized with 2.5% isofluorane in a mixture of N_2_/O_2_ (70:30) via a facial mask.

### Microdialysis Implantation

Microdialysis CMA7 metal free catheters (CMA microdialysis AB, Solna, Sweden) with molecular weight cutoffs of 6 kDa were sterilized and inserted into the brain of healthy rats. Sterile surgical procedures were used during the implantation of the microdialysis probes. The rats were deeply anesthetized and positioned in a stereotaxic instrument. The head was shaved and the skull was incised coronally between the ears, along the interaural line. Three small holes were created in the skull using a micro drill. In two holes, plastic screws were inserted while in the third hole a cannula guide was placed. The screws and the guide were fixed on the skull with dental glue and then the muscles and the skin were sutured. Following the surgery, the animals were housed individually and received doses of buprenorphine 0.05 mg/kg every 12 hours for 48 hours. Typically, the MR experiments took place 24 hours after the surgery.

### *In vivo* setup

The rat was positioned in prone position and the microdialysis was placed into the guide reaching the targeted region of the brain. A 8 mm surface dual channel-coil (Doty Scientific, Columbia, South Carolina) was placed over the microdialysis probe on the rat head. MRI T_1_ weighted scans (Flash sequence, Repetition Time 75.51 ms, Echo Time 2.6 ms, 6 averages, acquisitions time 1.25 min, matrix size 256 × 256, slice thickness 1 mm, Field of View 35 × 35 mm) were performed to assess the well-positioning of the microdialysis probe and the effective release of Dotarem^®^ in the cortical region. The outlet tube of the microdialysis probe was inserted into the microcoil tube to analyse the dialysate by MR Spectroscopy. The microdialysis probe was infused with a buffer solution (PBS in D_2_O containing 3 mM Dotarem^®^ 3 mM) under a flux of 500 nL/min.

### MR Experiments

^1^H experiments, *in vitro* and *in vivo*, were performed on a Bruker Biospec 12-cm bore 7.0 T using Paravision 6.0 software. A series of spectra (Single-pulse sequence, Repetition Time 1145 ms, 200 averages, acquisition duration 511.2 ms, spectral resolution 1.00 Hz/points, excitation pulse length 7.5 μs, flip angle 35°, excitation pulse power 250 mW, acquisition time 3.82 minutes, water suppression pulse, decoupling pulse) was acquired to check the metabolic evolution of dialysate.

^13^C experiments on U-87 cells were performed on a Bruker Biospec 12-cm bore 4.7 T using ParaVision 6.0 software. A series of spectra (Single Pulse sequence, Repetition Time 501.3 ms, 2000 averages, acquisition duration 500.0 ms, spectral resolution 1.00 Hz/points, excitation pulse length 10 μs,flip angle 50°, excitation pulse power 125 mW, acquisition time 16.71 minutes, decoupling pulse) was acquired to check the metabolic evolution of the dialysate.

### High-field analysis of dialysates

**Figure 5:**
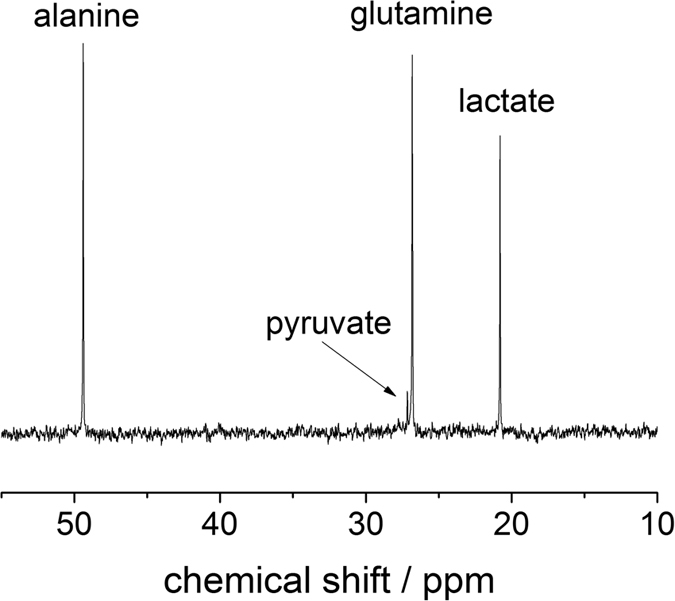
^13^C spectrum of the *in vitro* dialysate (dialysis experiment performed at B_0_ = 4.7T) detected at B_0_ = 11.7 T.

**Figure 6:**
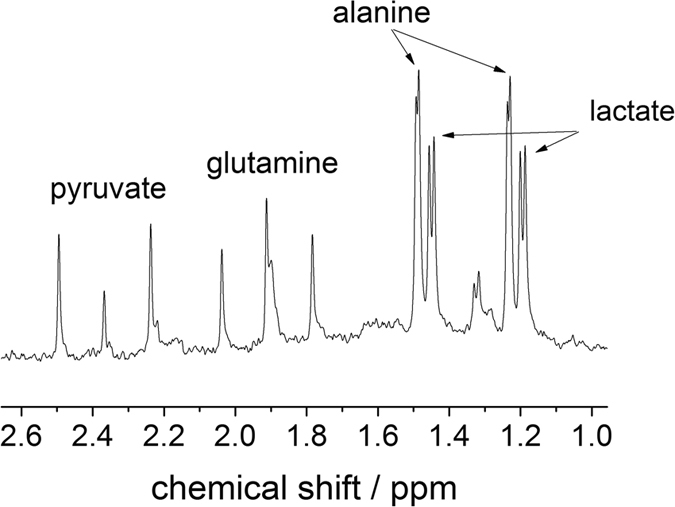
^1^H spectrum of the *in vitro* dialysate (dialysis experiment performed at B_0_ = 7T) detected at B_0_ = 11.7 T.

**Figure 7:**
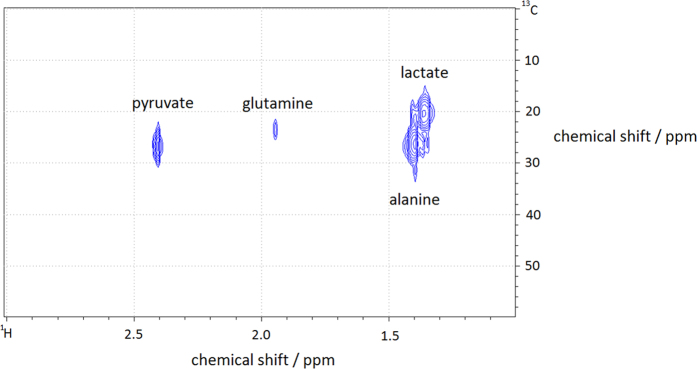
HSQC (Heteronuclear Single Quantum Correlation) spectrum of the *in vitro* dialysate (dialysis experiment performed at B_0_ = 7T) detected at B_0_ = 11.7 T to confirm the existence of ^13^C labeled metabolites.

**Figure 8:**
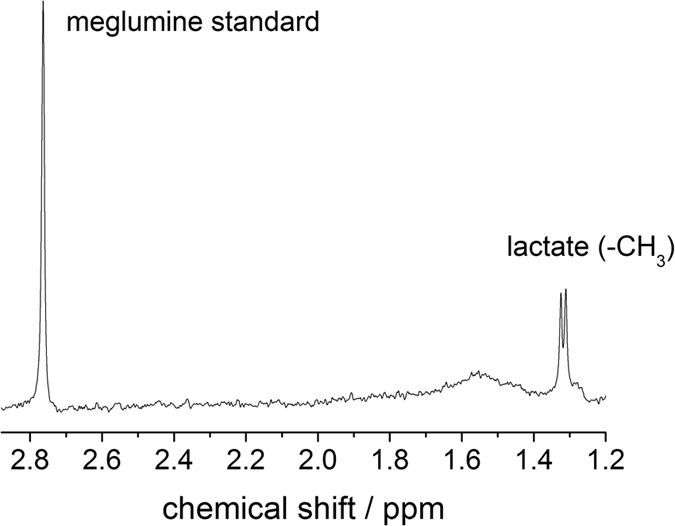
1H spectrum of the *in vivo* dialysate detected at B_0_ = 11.7 T.

High spectral resolution NMR experiments were performed on a Bruker DPX 500 MHz equipped with a BBI probe to confirm metabolites detection in MRI scanner. ^1^H spectra were obtained at 500.13 MHz through single pulse sequences (zg sequence, 128 scans, TD 16k, D1 1s) and single pulse with water suppression sequences (zgpr, 128 scans, TD 16k, D1 3s). ^13^C spectra were obtained at 125 MHz through single pulse excitation with power gate decoupling sequences (zgpg sequence, 128 scans, TD 16k, D1 1s). 2D HSQC ^1^H/^13^C acquisition was performed to determine the metabolites originating from enriched ^13^C pyruvate. We would like to note, that the observed metabolites in the high- field spectra depicted in Figs 5, 6, 7, 8 may have a different concentration than in the microcoil experiments since the spectroscopy experiment was run with the complete dialysate instead of measuring small portions of it.

### Ethical approval.

****Experiments were carried out following the INSERM (Institut National de la Santé et de la Recherche Médicale) guidelines regarding the fair treatment of animals with approval of the Comité d’Ethique en Expérimentation Animale de Bordeaux.

## Additional Information

**How to cite this article**: Glöggler, S. *et al*. *In vivo* online magnetic resonance quantification of absolute metabolite concentrations in microdialysate. *Sci. Rep.* **6**, 36080; doi: 10.1038/srep36080 (2016).

**Publisher’s note:** Springer Nature remains neutral with regard to jurisdictional claims in published maps and institutional affiliations.

## Figures and Tables

**Table 1:** Probe characteristics.

Probe	Frequency/MHz	Sample Volume/μL	Sensitivity^a^ at 14.1 T/nmol.s^½^	Linewidth/Hz	Unloaded Q-factor	B_1_/P^0.5b^/mT.W^−1/2^
^1^H-optimized	300	1.0	12.7	15.0 (0.07 ppm)	50	0.6
^13^C-optimized	50	1.0	14.6	3.5 (0.05 ppm)	100	3.7

a) Normalization to 14.1 T (According to ref. [25] equation 5 and Table 1): nLOD = 3n_s_ Δt^1/2^ SNR^-1^ (ΔB_0_)^7/4^ (ns: number of spins, Δt: effective acquisition time, SNR: measured signal-to-noise ratio, ΔB_0_: difference between utilized magnetic field and 14.1 T).

b) Probe efficiency: Based on the reciprocity principle^2^,^25^, the probe efficiency B_1_/P^0.5^ (where B_1_ is the RF magnetic field for a 360° pulse at a given transmitter power P) is directly proportional to the detection sensitivity of a probe.
